# Direct Observation of Strand Passage by DNA-Topoisomerase and Its Limited Processivity

**DOI:** 10.1371/journal.pone.0034920

**Published:** 2012-04-09

**Authors:** Katsunori Yogo, Taisaku Ogawa, Masahito Hayashi, Yoshie Harada, Takayuki Nishizaka, Kazuhiko Kinosita

**Affiliations:** 1 Department of Physics, Faculty of Science and Engineering, Waseda University, Tokyo, Japan; 2 Yasuda “On-chip Molecular Cell Phenomics” Project, Kanagawa Academy of Science and Technology (KAST), Kawasaki, Japan; 3 Institute for Integrated Cell-Material Sciences (iCeMS), Kyoto University, Kyoto, Japan; 4 Department of Physics, Gakushuin University, Tokyo, Japan; Institut Pasteur, France

## Abstract

Type-II DNA topoisomerases resolve DNA entanglements such as supercoils, knots and catenanes by passing one segment of DNA duplex through a transient enzyme-bridged double-stranded break in another segment. The ATP-dependent passage reaction has previously been demonstrated at the single-molecule level, showing apparent processivity at saturating ATP. Here we directly observed the strand passage by human topoisomerase IIα, after winding a pair of fluorescently stained DNA molecules with optical tweezers for 30 turns into an X-shaped braid. On average 0.51±0.33 µm (11±6 turns) of a braid was unlinked in a burst of reactions taking 8±4 s, the unlinked length being essentially independent of the enzyme concentration between 0.25–37 pM. The time elapsed before the start of processive unlinking decreased with the enzyme concentration, being ∼100 s at 3.7 pM. These results are consistent with a scenario where the enzyme binds to one DNA for a period of ∼10 s, waiting for multiple diffusional encounters with the other DNA to transport it across the break ∼10 times, and then dissociates from the binding site without waiting for the exhaustion of transportable DNA segments.

## Introduction

Type II DNA topoisomerases let one segment of double-stranded DNA pass through another without leaving a permanent cut in either of the segments, thus resolving DNA entanglements in cells [1]–[4]. The multi-subunit enzyme (dimeric in eukaryotes) is twofold symmetric, and the interface between the two halves consists of three gates (N-, DNA-, and C-gates) that can open and pass a DNA duplex [5]. In the current view, the enzyme first binds one DNA segment, termed the G (gate) segment, at the DNA-gate to cleave the double helix while keeping the resultant 5′-ends bonded to the catalytic tyrosines on each side of the DNA gate. When another DNA segment (T or transport segment) comes, the enzyme transports it sequentially through the three gates in ATP-dependent reactions, resulting in the passage of the T segment across the G segment. Passages of multiple T segments seem to be allowed before the religated G segment eventually leaves the enzyme. How the apparent processivity is regulated, as well as the precise control of T segment passage without dissociation of the G segment from the enzyme or of the dimeric enzyme itself, are yet to be clarified.

The action of type II topoisomerases has been studied at the single-molecule level, with DNA tethering a micron-sized bead to a surface [6]–[9]. Rotating the bead introduced supercoils (plectonemes) in a single DNA tether, or a braid in a dual DNA tether, both resulting in lowering of the bead. In the presence of topoisomerase, the bead suddenly floated upward, indicating resolution of the entanglements by topoisomerase. These early studies suggested that the enzyme is highly processive. Charvin et al. [7], for example, reported that 40- or 80-turn braids they formed were completely unlinked in one burst of reactions in all cases examined. Such a high processivity would imply that, throughout the burst, both of the two DNA molecules can interact with the (single) enzyme molecule simultaneously. Whether the DNA geometry beneath the bead would allow these simultaneous encounters was not directly confirmed in the previous studies.

A particular concern is that a magnetic bead in a magnetic field assumes a certain orientation peculiar to each bead (hence the bead can be rotated by rotating the field). Thus, when two DNA molecules of identical lengths are attached to one magnetic bead and the bead is pulled by a magnet, one of the two DNA molecules must be slack in almost all cases, becoming taut only after a variable amount of braiding and even then the tension will be different between the two DNAs. Complete unwinding of a loose braid will be difficult to discern unless one observes the DNAs directly. In the case of a single DNA tether, disappearance of supercoils is directly reflected in the bead movement, but supercoils are highly dynamic and thus their geometry is not well defined.

**Figure 1:**
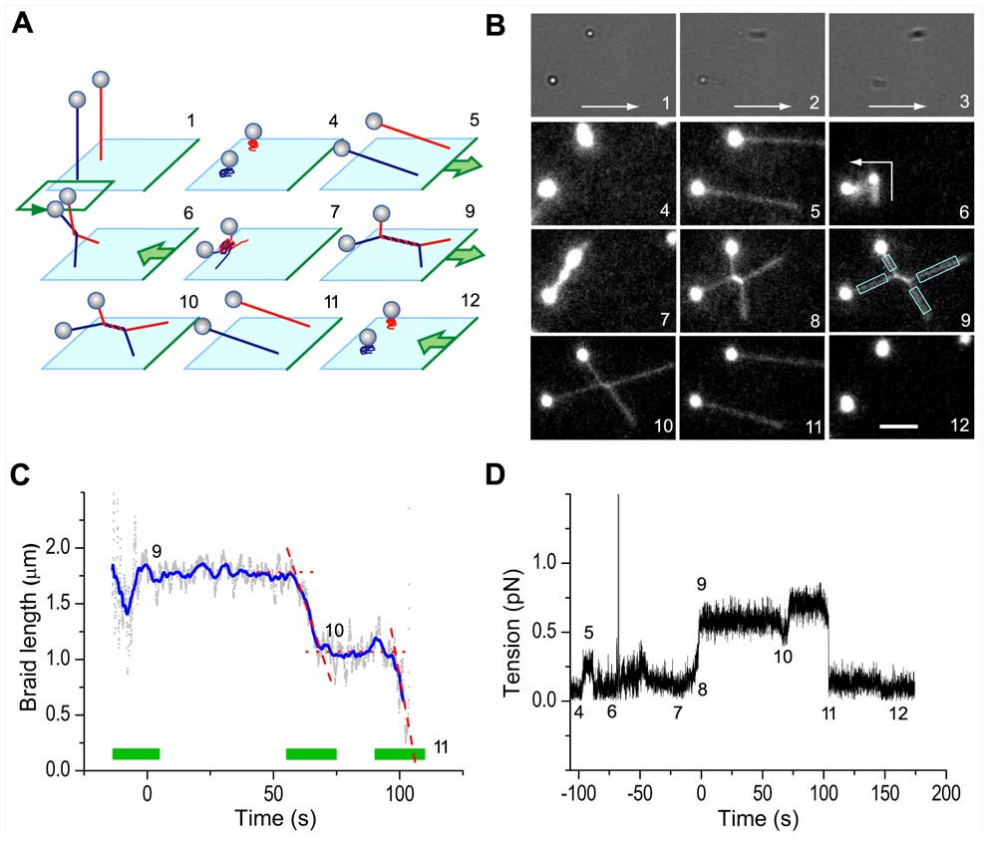
Experimental design. (**A**) Schematic diagrams showing the experimental procedure where the floating ends of the two DNAs were manipulated with optical tweezers while their root positions were controlled by stage movement. Numbers correspond to those in **B**–**D**. (**B**) Snapshots of bright-field (1–3; sequential frames) and fluorescence images (4–12; averaged over 30 frames = 1 s). Arrows in 1–3 show the direction of stage movement. Rectangles in 9 show the regions where the DNA images were fitted with a line to estimate the braid length. The scale bar in 12 shows 5 µm (57.5 pixels). (**C, D**) Time courses of the braid length (**C**) and the DNA tension sensed by the lower-left bead in **B** (**D**). Time 0 is the end of stage movement. After the processive unbraiding at ∼60 s, we slightly increased the tension at ∼70 s. Gray dots in **C** were calculated on images averaged over 30 frames, and further averaging over 120 frames (4 s) shown in blue. Red broken lines show the way the burst time was estimated. Green horizontal bars in **C** indicate portions shown in Viedo S1. The total tension in **D** represents (*T*_x_^2^+*T*_y_^2^)^1/2^, and thus noise, converted to positive values, dominates over the actual tension when the latter is below the noise level. The tension is essentially zero at stages 4 and 12.

Here we visualized DNA with fluorescence staining, and made a braid between a pair of DNA molecules by manipulating each DNA independently. The braid was at the center of an X-shaped DNA pair (Figure 1) with crossing angles of ∼90°. Thus the two DNA molecules could meet only within, or near an end of, the braid. With this clearly defined geometry, we found that the processivity of human topoisomerase IIα (hereafter referred to as topo IIα) is on average ∼10 braid turns, limited by a finite burst reaction time of ∼10 s after which the enzyme presumably dissociates from DNA.

## Results

### Direct Observation of Unbraiding Reaction

To braid DNA, we made a sparse lawn of λ-phage DNA (one molecule per 10^2^–10^3^ µm^2^) by attaching one end of the 16-µm DNA to a glass surface. We then slowly infused topo IIα several times to establish a pM concentration of free and active enzyme. The final infusion included SYBR Gold, a fluorescent dye for DNA staining, and polystyrene beads (0.92 µm) to be attached to the floating ends of DNA for manipulation (Figure 1A*1*). We selected in a bright-field image a relatively isolated pair of beads that were appropriately separated (5–15 µm) and undergoing tethered diffusion. Each bead was confirmed to be tethered by one DNA molecule, by holding the bead in an optical trap and moving the microscope stage in different directions until the bead escaped from the trap (Figure 1B*1–3*). We then turned on fluorescence excitation, stretched the DNA pair to confirm the absence of extra DNA segments (Figure 1A*5*, B*5*), and manipulated the two beads with dual-beam optical tweezers to braid the two DNA molecules tethering the beads (Figure 1A*6*, B*6*). We wound one DNA around the other 30 times, unless stated otherwise. The resultant braid contained 29.5 turns, because we stopped when the bead came back to the original position, but we call this a 30-turn braid for simplicity throughout this paper. In most cases winding was counterclockwise as viewed from above. Occasional clockwise trials did not show an appreciable difference (see figures below), and thus we ignore the sense of winding in this paper. After winding, we lowered the two beads to 1±0.5 µm (bead center) above the glass surface, and adjusted the bead positions such that the two DNA molecules would eventually form an X shape with ∼90° crossings (Figure 1A*7*, B*7*). We then displaced the stage until the DNA pair formed an X shape (Figure 1A*9*, 1B*9*) and the tension judged from the bead displacement was 0.95±0.26 pN (mean±s.d. for 85 DNA pairs; ranged 0.44–1.7 pN). The stage movement (∼10 µm) typically took 10–20 s, the worst case being ∼60 s (no unbraiding reaction followed in this case) and three more cases being ∼30 s. We take the end of the stage movement as time 0.

Unbraiding reactions after time 0 were detected as a sudden decrease in the braid length (Figure 1C, ∼60 s and ∼100 s; Video S1) and a slight drop in DNA tension (Figure 1D*10*). In the example shown in Figure 1, unbraiding occurred in two bursts, with a decrease of braid length of ∼0.7 µm in ∼10 s and ∼1.0 µm in ∼8 s. From the relationship between the braid length and the number of braid turns obtained in the absence of topo IIα (Figure S3), which depends largely on the crossing angles between the two DNA molecules and to some extent on the tension, we estimate that the burst lengths above correspond to the releases of ∼13 and ∼15 turns, respectively (the sum slightly differs from 30 because of imprecision in the calibration and the burst lengths). After the first burst, we increased the tension slightly by stage movement (Figure 1D*10*), to confirm that the pause in unbraiding was not due to the reduction in tension (such a maneuver was attempted in nine cases at 3.7 pM topo IIα, all failing to restart unbraiding). The second burst resulted in complete unbraiding, as seen in the fluorescence image (Figure 1B*11*), and the tension dropped to almost 0 (Figure 1D*11*). The residual small tension due to elongated DNA tether became completely negligible when the stage was moved back (Figure 1D*12*). Unless the topo IIα concentration, [topo IIα], was high (>∼10 pM), complete unbraiding as in Figure 1 was rare: five out of 40 DNA pairs at 3.7 pM topo IIα and none below. In cases where unbraiding was incomplete at 300 s, we terminated observation and mechanically undid the braid with the optical tweezers, in the absence of fluorescence excitation, to count the number of turns that remained in the braid. The remaining number was consistent with the braid length in most cases, with exceptions where the mechanical count was obviously smaller (Figure S4). We ascribe the latter to topo IIα-induced unbraiding during mechanical unwinding that took time (the presence of a braid had to be checked after each turn).

### Size of Unbraiding Bursts

**Figure 2:**
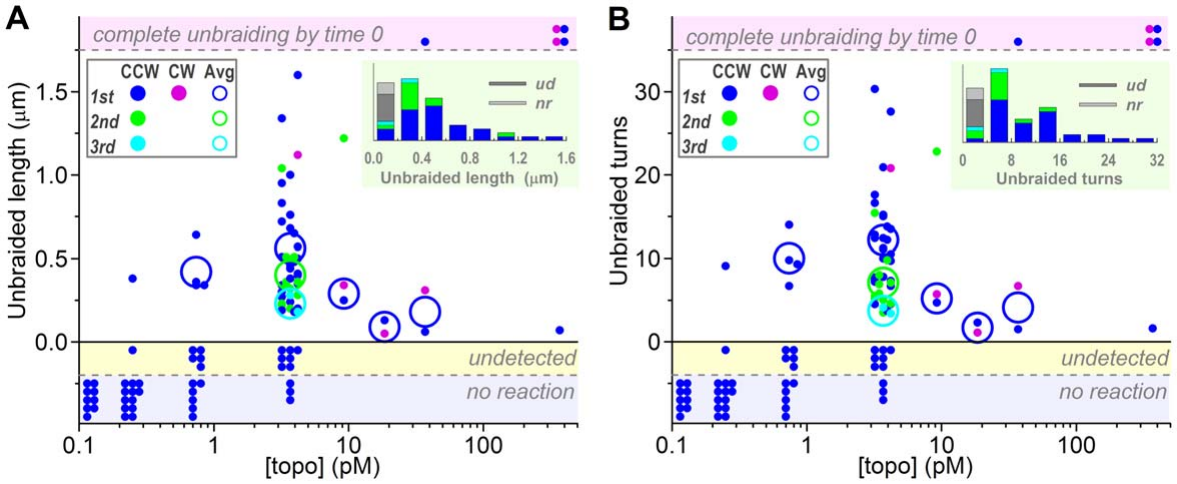
Unbraiding burst sizes. (**A**) Burst sizes estimated from the braid length in fluorescence images. (**B**) Burst sizes in terms of braid turns; the braid length was converted to braid turns using the calibration equation described in Figure S3. For both panels, dots show individual observations, plotted in two or three lines for clarity. Large circles show averages for the first (blue or purple), second (green), and third (cyan) bursts, counterclockwise (CCW) and clockwise (CW) braids not distinguished. The averages are for the data in which unbraiding was confirmed as a change in braid length (dots in the central white zone). Dots in the top shaded zone indicate cases where a braid completely disappeared by the time the tension was set up (time 0). The shaded zone below vertical zero shows cases where a length change was undetected but mechanical unwinding at the end (300 s) revealed remaining braid turns of less than 30. The bottom shaded zone is for no reaction cases where the braid number remained 30 until 300 s as confirmed by mechanical unwinding. Insets at top right show histograms of unbraided length/turns at 3.7 pM topo IIα; *ud*, undetected; *nr*, no reaction.

At 3.7 pM topo IIα, the length of a braid released in one burst averaged 0.51±0.33 µm (s.d. for 43 bursts), corresponding to the number of unbraided turns of 10.5±6.2 (Figure 2). The second and third reactions in multiple burst cases resulted in progressively smaller burst sizes (green and cyan in Figure 2): the first bursts averaged 0.56±0.35 µm or 12.2±6.4 turns (*n* = 30) whereas the second bursts 0.40±0.24 µm or 7.0±3.3 turns (*n* = 11) and the thirds 0.23±0.07 µm or 3.7±0.5 turns (*n* = 2). At lower [topo IIα]s, the unbraiding reactions became rare, but the burst size did not change appreciably, suggesting that each burst was catalyzed by one topo IIα molecule. At 3.7 pM topo IIα, the braid length at time 0 was sometimes clearly shorter than that expected for 30 turns (Figure S4), indicating partial unbraiding during the tension application by stage movement or possibly earlier. The premature unbraiding was more extensive at higher [topo IIα]s, where, in most cases, the braids that had remained at time 0 were already short and disappeared in one short burst. At 370 pM, no braid remained by the time ( = 0) the tension was to reach ∼1 pN in four out of five cases (Figure 2, *top shaded zone*), and winding for 100 turns did not improve this situation. Presumably, the premature unbraiding occurred as rapid succession of multiple bursts rather than one burst that encompassed the whole braid.

### Frequency of Unbraiding Bursts

**Figure 3:**
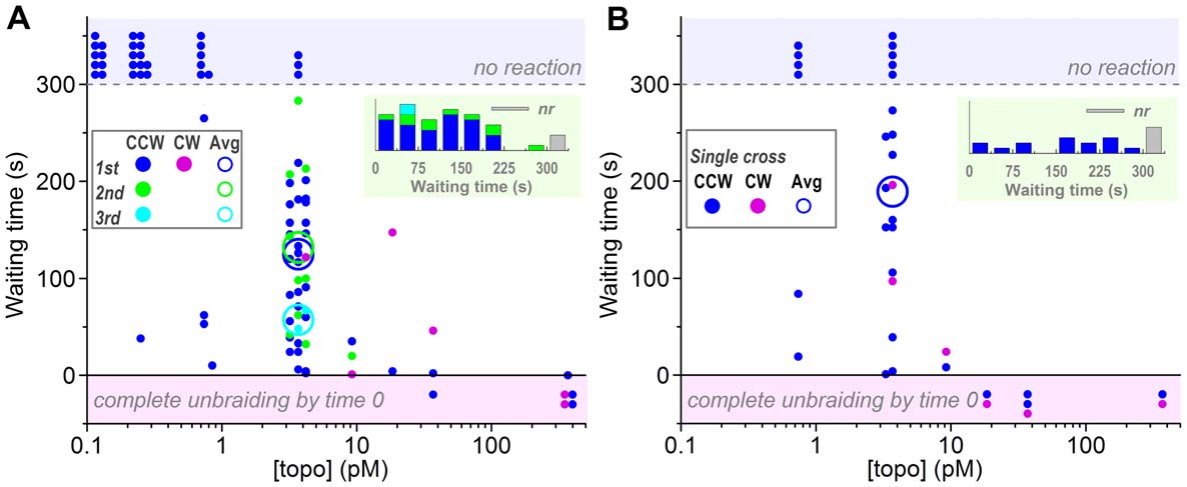
Waiting times before an unbraiding reaction starts, measured from time 0 (for first bursts) or from the preceding burst (second and third bursts). Observations were terminated at 300 s. (**A**) Experiments with a 30-turn braid shown in Figure 2. (**B**) Experiments with a single-cross braid, attempted after an experiment in **A** when the DNA pair remained intact. See Figure 2 for symbols. Note that the averages are shown only for [topo IIα] = 3.7 pM, because the cases of no reaction (top shaded zone) or premature unbraiding (bottom shaded zone) introduce too much ambiguity. In the averages for the first bursts, the waiting times for the no reaction cases are taken as 300 s, leading to underestimates. Second and third bursts are also underestimated because of the 300-s limitation in the total observation time. Insets at top right show histograms of waiting times at 3.7 pM topo IIα; *nr*, no reaction.

In contrast to the burst size which was essentially independent of [topo IIα], the waiting time before an unbraiding burst, whether from the tension setup to the first unbraiding or between two successive unbraiding bursts, decreased greatly as [topo IIα] was increased (Figure 3A). Precise quantitative analysis of the waiting times is not possible because times longer than 300 s were not measured, because short waiting times involve the uncertainties of 10–20 s needed for the stage movement, and because premature unbraiding at high [topo IIα]s could not be timed. The trend in Figure 3A, however, suggests that the waiting time is inversely proportional to [topo IIα], the relation expected for the scenario where binding of one topo IIα molecule initiates each unbraiding burst. The average waiting time at 3.7 pM topo IIα of ∼100 s (excluding no-unbraiding cases and thus being an underestimate) indicates an effective topo IIα binding rate of ∼3×10^9^ M^−1^s^−1^. A surface plasmon assay [10] has shown a rate of human topoisomerase IIα binding to 166 bp (56 nm) DNA of 9×10^6^ M^−1^s^−1^ at 20°C, while binding of *Saccharomyces cerevisiae* DNA topoisomerase II to 40 bp (14 nm) DNA assessed through fluorescence anisotropy [11] has given 1×10^9^ M^−1^s^−1^ at 30°C. Our value above would be in between for the target DNA size of 1–2 µm (braid length).

When the DNA pair remained intact (not detached from the bead or glass surface and no extraneous DNA on beads) after an unbraiding assay and subsequent mechanical unbinding, we made a single cross (a half-turn braid that we would call one turn in this paper) with the same DNA pair and waited until topo IIα undid the cross or 300 s had passed. Such trials were made up to three times for the same pair. The waiting times for single crosses (Figure 3B) were grossly similar to those for 30-turn braids (Figure 3A), indicating that both topo IIα and DNA survived the rather long (but low intensity) fluorescence excitation. The waiting times for single crosses, however, are longer on average, particularly when the no reaction cases are taken into account. This could be due to the long fluorescence excitation, but a more likely explanation is the smaller target size for topo IIα binding. In a DNA cross, the two DNAs slide past each other by thermal motion, and binding of a topo IIα molecule within the thermally overlapping zone will be effective. The effective overlap is on the order of 0.2 µm (Text S1), several times smaller than the length of a 30-turn braid.

### Duration of Unbraiding Bursts

We estimated the duration of each unbraiding burst by drawing three straight lines, as shown in Figure 1C, to define the beginning and end of the burst. We did not find obvious [topo IIα] dependence, and obtained 8±4 s (ranged 2–22 s; *n* = 48, ambiguous cases omitted) for the burst duration. In each burst, strand passages took place at a rate of ∼1 s^−1^. Burst times of ∼10 s or less have been reported for relaxation of supercoiled DNA [6].

## Discussion

In summary, our results show that human topoisomerase IIα unlinks a DNA braid of crossing angles ∼90° and under ∼1 pN of tension in bursts of ∼0.5 µm or ∼10 turns, each taking ∼10 s. One topoisomerase molecule seems to mediate each burst, by staying on one strand of DNA and allowing multiple passages of the other DNA for a period of ∼10 s. These results are largely consistent with previous single-molecule unlinking assays [6]–[9], except that our [topo IIα] is an order of magnitude lower for similar reaction kinetics. We tried to minimize possible loss/damage of topo IIα on surfaces during dilutions and infusions, which may partly explain the difference.

**Figure 4:**
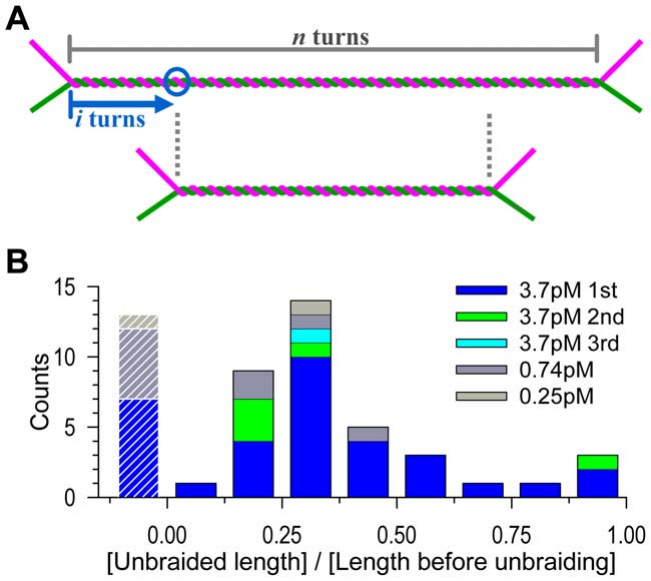
Distribution of unbraiding burst sizes. (**A**) The expected length of unbraiding. If a topo IIα molecule binds at *i*-th turn (circle) from an end of a braid of *n* turns, and if the topo IIα stays and remains active for a sufficient period, then the braid length will become *n* – 2*i*, or the unbraiding length will be 2*i*, because the two DNA segments forming the braid can freely slide against each other to keep the braid center at the same position. For random binding, *i* is anywhere between 0 and *n*/2, and thus the unbraiding length 2*i* will distribute equally between 0 and *n*, averaging *n*/2. Thermal motion of DNA will increase the unbraiding length by several turns (Text S1). (**B**) Observed distribution. The unbraiding lengths in Figure 2A are normalized by the length before unbraiding (corresponding to *n* in **A**). Data with an initial length greater than 1 µm have been selected and analyzed. The leftmost bars with stripes represent cases where unbraiding was undetected in the length assay; the normalized unbraiding lengths for these data should be less than 0.25 (Figure S4).

All single-molecule assays including the present study have shown processive action of topoisomerase II for a duration of the order of 10 s. A recent study with fluorescence energy transfer [12] has shown that, once topoisomerase II (*Drosophila*) binds a DNA segment, it undergoes many MgATP-dependent cycles in which the two ends of the cleaved G-segment DNA are widely separated for ∼1 s and then come close (and possibly religated) for another ∼1 s. Such a behavior, observed in the absence of a T segment to be transported, would warrant processivity in that topoisomerase II residing on a G segment is ready to transport through it many other T segments when they come by diffusion. In contrast to the report by Charvin et al. [7], however, total unbraiding in one burst was rare in our experiments. We have no definite explanation, except to point out that they worked at somewhat lower ionic strength (50 mM KCl+50 mM NaCl compared to our 50 mM KCl+100 mM NaCl) which would favor processivity [13], that their DNAs were crossed at shallower angles and attached to one bead (one of the two DNAs might have been slack, precluding the detection of complete unbraiding), that they worked at a higher [topoisomerase] of *∼*30 pM, and that their enzyme was of *Drosophila melanogaster*. For a single topoisomerase molecule to completely undo a braid in an X-shaped DNA pair with 100% success, the enzyme would have to somehow move along the DNA not randomly but in the direction toward the braid center (see Figure 4A). DNA, however, is basically symmetric, and topoisomerase itself is twofold symmetric [5]. Or, the topoisomerase may catch up a shrinking braid by preferential binding to DNA crossovers [14], but this would require dissociation before each rebinding.

Our results here suggest that the processive action of topo IIα is limited in time. If a topo IIα molecule on a DNA segment can execute unlimited cycles of strand passages, the reaction would stop when an end of the braid reaches the topo IIα binding site (Figure 4A). On average, then, 15 turns would be unbraided out of the initial 30 turns, and thermal sliding of the DNAs against each other (Text S1) would allow a few more turns to be released. The experimental average, in contrast, was 9.6 turns for the 48 first bursts at [topo IIα] = 3.7 pM (Figure 2B), the undetected cases being included. It seems that topo IIα does not always wait until a braid end comes. Figure 4A also shows that the distribution of burst sizes would be uniform if topo IIα stays and remains active for an indefinite time. Experimental distribution (Figure 4B), although the statistics is not good enough to allow the rigorous analysis suggested [15], is biased toward short bursts, again pointing to a limited activity; note that very short unbraiding bursts were likely unnoticed in our length measurement whereas some of the long ones may actually have been effected by two topo IIα molecules. The implication is that, after the average burst period of ∼10 s, topo IIα dissociates from DNA (or possibly lapses into an inactive state), rather than waiting for the exhaustion of transportable DNA. Perhaps, visiting many different DNA entanglements is physiologically more advantageous than staying long on one segment.

## Materials and Methods

### Materials

We purchased recombinant human topoisomerases IIα (NCBI RefSeq NP_001058.2, unmodified, 340 kDa as homodimer) from Amersham Pharmacia (USB Product No. 78303Y) and confirmed its stock concentration reported by the supplier of 0.4 mg/mL by Bradford assay with BSA as standard. We confirmed in bulk assays (Figure S1) that the enzyme relaxed negatively supercoiled pBR322 plasmid DNA (Nippon Gene) with kinetics similar to or slightly faster than reported [13], that the activity was blocked by the omission of ATP or by the inhibitor ICRF-193 (Enzo Life Sciences) as described [16], and that the enzyme introduced a double-stranded break in the plasmid DNA in the presence of etoposide (TopoGEN) to produce linear DNA [17]. For dilution of the enzyme stock, we used non-adsorbing tips and tubes, discarding first dilutions. We labeled ends of unmethylated λ-phage DNA (48.5 kb, Promega) with single digoxigenin and single biotin through 12-mer primers. Carboxylated polystyrene beads (0.92 µm, Bangs) were biotinylated and streptavidin was bound [18].

### Observation Chamber

A ∼10 µL flow chamber was made of two cover slips, methanol cleaned and collodion coated [19]. Infusions (in chamber volumes) and incubations were made as follows: 1 volume of base buffer (10 mM Tris pH 7.9, 100 mM NaCl, 50 mM KCl, 5 mM MgCl_2_, 0.1 mM EDTA); 5 min; 1 vol. 20 µg/mL anti-digoxigenin antibody (Roche) in base buffer; 5 min; 3 vol. 1 mg/mL N,N-dimethylated casein (Sigma) and 0.2% Tween-20 in base buffer for surface blocking; 5 min; 3 vol. topo buffer (3 mM ATP, 5 mM DTT, 0.1 mg/mL dimethylated casein, 0.2% Tween-20 in base buffer) for washing; 1 vol. 5 pM labeled DNA in topo buffer; 15 min; 5 vol. topo buffer containing a desired amount of topoisomerase; 1 vol. the same solution containing ∼10^7^ streptavidin beads/mL and 800,000-fold dilution of SYBR Gold (Molecular Probes). Finally the chamber was sealed with grease. All solutions were prepared with degassed water. Bulk relaxation activity was not affected by SYBR, casein, and Tween-20 (Figure S1). We also checked decatenation activity with kinetoplast DNA as substrate (http://www.topogen.com/A_Decatenation.pdf). The decatenation activity was unaffected by the presence of SYBR Gold at 10,000-fold dilution (34±7 arbitrary unit, mean±s.d. for *n* = 4) or 0.2% Tween-20 (31±2, *n* = 2) compared to control (37±6, *n* = 5).

### Microscopy and Manipulation

Fluorescence image of DNA and bright-field image of beads were simultaneously observed on an inverted microscope (IX 70, Olympus) equipped with dual-beam optical tweezers (1064 nm) [20]. Fluorescence was detected with an intensified (VS4-1845, Videoscope) CCD camera (R300, Dage-MTI), and beads with another CCD camera (R300), both at 30 Hz. Two beads tethered to a pair of DNA molecules were manipulated by moving the trapping beams manually or under computer control with which one DNA could be wound around the other for 30 turns in 7 s. The other ends of the DNAs were manipulated by moving the motorized sample stage. Stiffness of the optical traps was estimated from the (Gaussian) distribution of the positions of a trapped bead with a fast-shutter (1 ms) camera (FC300M, Takenaka System) at a reduced laser intensity. Observations were made at 25°C.

### Analyses

Centroids of bead images were calculated [21] in real time with Video-Savant software (IO Industries), which also recorded digitized images on a hard disk. Length of a DNA braid was estimated from the recorded images, after averaging over 30 frames (1 s), by fitting each of the four branches of the X-shaped DNA pair with a line with weights proportional to the fluorescence intensity (Figure 1B *9*). Static images (also averaged over 30 frames) were also fitted with lines by eye, after magnification, giving similar results. Calibration in the absence of topo IIα (Figure S3) indicates an average precision of ±0.12 µm (±1.4 pixel).

## Supporting Information

Figure S1 **Bulk supercoil relaxation activity of topo IIα.** The enzyme at the indicated concentration was incubated with 5 nM pBR322 plasmid (negatively supercoiled) and 1 mM ATP (unless indicated otherwise) for the indicated time at 37°C in 20 µL of buffer B for bulk assay or buffer M for microscopy. Buffer B was the base buffer described in the main text (10 mM Tris pH 7.9, 100 mM NaCl, 50 mM KCl, 5 mM MgCl_2_, 0.1 mM EDTA) containing, in addition, 0.1 mg/mL BSA. For buffer M, the base buffer was supplemented with 5 mM DTT, 0.1 mg/mL dimethylated casein, 0.2% Tween-20, and 800,000-fold dilution of SYBR Gold. The reaction was started by the addition of 1 µL of the enzyme diluted in buffer B containing 0.5 mM DTT, and terminated by the addition of 2 µL of 5% SDS and 100 mM EDTA. Samples were mixed with 2 µL of gel loading buffer (50% glycerol, 0.9% SDS, 0.05% Bromophenol Blue), heated at 70°C for 2 min, subjected to electrophoresis in a 1% agarose gel in 90 mM Tris-borate pH 8.4 and 2 mM EDTA, and stained with 10,000-fold dilution of SYBR Gold. (**A**) Time courses of relaxation. (−)SC, negatively supercoiled plasmid; relaxed, relaxed circular plasmid. (**B**) Effects of topoisomerase-specific drugs. ICRF-193, a bis(2,6-didioxopiperazine) derivative, is a potent inhibitor of type II topoisomerase with 50% inhibition at ∼2 µM [16]. Etoposide inhibits religation of DNA, producing double-strand breaks irrespective of the presence of ATP [17]. Assays were made as in **A** for the reaction period of 30 min. Samples with etoposide were incubated, prior to electrophoresis, with 200 µg/mL proteinase K at 45°C for 30 min to digest topo IIα. Linear DNA marker was generated by digesting pBR322 by EcoRI. The yield of linearized DNA at 200 µM etoposide is below 10% at the enzyme/pBR322 molar ratio of five under similar conditions [17].
(TIF)

Figure S2 **A geometrical model for a DNA braid.** The model has been described by Charvin et al. [7] for the case of a symmetric braid (*Δα* = 0). Here, the angles between the DNA and braid axis are *α*+*Δα* (magenta) and *α*-*Δα* (green), which are assumed to be maintained through the braid. In the braid, the distances between the DNA axis and braid axis are taken as *R*+*ΔR* (magenta) and *R*-*ΔR* (green). The pitch *P* is then given as *P* = 2π(*R*+*ΔR*)/tan(*α*+*Δα*) for magenta DNA and *P* = 2π(*R* – *ΔR*)/tan(*α* – *Δα*) for green DNA. The two pitches must be the same in a braid, leading to *P* = 2π*R*cot*α*(1 - *Δα* ^2^/cos*α* ^2^) where terms higher than *Δα* ^2^ have been neglected. For a braid of (*n* – 1/2) turns (made by winding one DNA around the other for *n* turns as described in the main text), its length *l* is given by *l* = (*n* – 1/2)*P*. The effect of the asymmetry (*Δα*) is of the second order and is practically negligible, and thus *l*∼2π(*n* – 1/2)*R*cot*α* = 2π(*n* – 1/2)*R*tan(π/2 - *θ*/2), where *θ* = 2*α* is the angle between the two DNAs. This final result is the same as that by Charvin et al. [7], and the only merit of the calculation above is to show that asymmetry does not matter as long as *Δα*<<1 (radian). Note that the geometrical model would fail at *Δα*>45° where the magenta and green rods would overlap partially, although *R* here represents an entropic radius of DNA [22] which is much larger than the geometrical radius of ∼1 nm and thus the failure would not be a sudden one expected for solid ropes.
(TIF)

Figure S3 **Determinants of braid length.** Pairs of DNA were braided and the braid lengths estimated from fluorescence images as described in the main text, except that topoisomerase was not included in the medium. (**A**) Fluorescence images of a DNA braid of *n* = 30, showing dependence of the braid length on braid angles. (**B**) Proportionality between braid length and braid turns (*n* – 1/2) for *θ* around 90° (range 75°–95°). (**C**) Angle dependence of the braid length for *n* = 30. The lower horizontal axis is chosen to test the geometrical model in Figure S2. Open circles, tension between 0.7 and 1.4 pN; black circles, below 0.7 pN; gray circles, above 1.4 pN. Dashed line shows regression passing through the origin (geometrical model) for open circles, with *l* = 1.37tan(90° - *θ*/2) where *l* is the braid length in µm. A better fit is obtained if we allow the line to deviate from the origin (solid line), with *l* = 0.51+0.96tan(90° - *θ*/2). Deviation from the geometrical model (Figure S2) is expected for *θ*>90°, and the observed braid pitch of ∼50 nm (*l*/*n*), close to the persistence length of DNA, also suggests that bending the DNA may cost additional energy. (**D**) Tension dependence of the braid length (*n* = 30) for *θ* around 90° (range 79°–97°). (**E**) Braid lengths in **D** converted to those at *θ* = 90° by assuming the angle dependence shown in the solid line in **C**. Solid curve shows fit assuming that the normalized braid length depends on the tension *F* as *F* ^−3/4^ [7]: *l* = 0.90+0.63*F* ^−3/4^ where *F* is in pN. In the main text and in Figure S4 below, we estimate the braid turns *n* from the observed braid length *l* and tension *F* assuming this tension dependence and the solid line in **C**: *l* = [(*n* – 1/2)/(29.5·1.47)][0.51+0.96tan(90° - *θ*/2)][0.90+0.63*F* ^−3/4^] or *n* = 1/2+72*l*/{[0.53+tan(90° - *θ*/2)](1.43+*F* ^−3/4^)}. The root-mean-square deviation of the 58 measured braid lengths from this phenomenological equation (four data with tan(90° - *θ*/2)<0.5 excluded) is 0.12 µm, which is a measure of the reliability of the length estimates.
(TIF)

Figure S4 **Summary of individual unbraiding reactions.** Results from each experiment are shown in paired bars, the left bar showing the braid turns counted by mechanical winding/unwinding and the right bar showing the observed braid lengths which have been converted to the braid turns by the last equation in Figure S3. All the left bars are given the same height of 30 turns, the count in the initial mechanical winding. Note that the count at time 0 may have been smaller, due to premature unbraiding particularly at high [topo IIα]s. The magenta portions show the braid turns that remained at 300 s, estimated by mechanical unbraiding. Some of these may be underestimated, due to unbraiding by topo IIα during the process of mechanical unbraiding. The height of the bars on the right corresponds to the braid length at time 0. The height deviates from 30, because the determination of the braid length and the conversion to braid turns both involve errors, and because premature unbraiding took place at high [topo IIα]s. For the same reasons, the magenta portion in the left bar and the red portion in the right bar do not match precisely. “ud” indicates the cases where the mechanical count suggested unbraiding but a decrease in the braid length could not be detected reliably. Note that failure in detecting a length change can be inferred only when the initial length was apparently preserved; when unbraiding is detected both in the mechanical counts and length changes, there is always a possibility that an additional short unbraiding event(s) has taken place without being detected. CW, clockwise braid.
(TIF)

Video S1 **Unbraiding of a 30(29.5)-turn DNA braid in two bursts.** The movie consists of three portions (initial setup, first unbraiding burst, second burst resulting in complete unbraiding) as indicated by green horizontal bars in Figure 1C in the main text, with 1-s blackouts in between. The time displayed on the lower right matches that in Figure 1C. The white bar that appears at time 0 indicates 1.8 µm, the initial braid length. Note that the stage, and thus the DNA roots, were slightly moved to the right between ∼70 and ∼75 s to increase the tension (Figure 1D). The movie taken at 30 frames/s has been running-averaged for 30 frames, and the intensity adjusted to highlight DNA (bead images are saturated).
(AVI)

Text S1 **DNA fluctuations in a braid.**
(DOC)
