# Supplementary figures and images for: Direct Observation of Strand Passage by DNA-Topoisomerase and Its Limited Processivity
Source: PLoS One. 2012 Apr 9;7(4):e34920. doi: 10.1371/journal.pone.0034920 (PMC3322154; doi:10.1371/journal.pone.0034920)

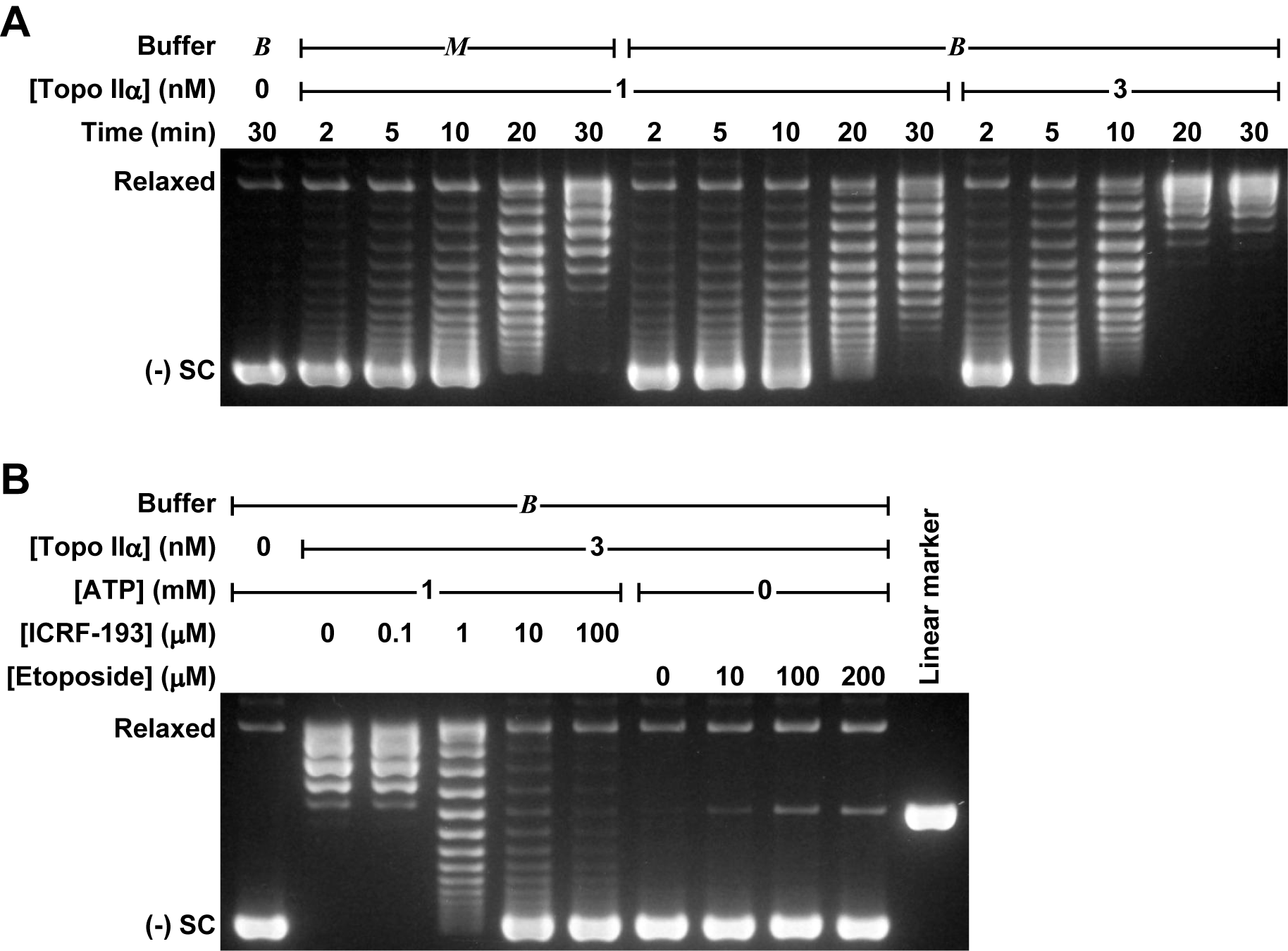

Supplement: Figure S1 — Bulk supercoil relaxation activity of topo IIα. The enzyme at the indicated concentration was incubated with 5 nM pBR322 plasmid (negatively supercoiled) and 1 mM ATP (unless indicated otherwise) for the indicated time at 37°C in 20 µL of buffer B for bulk assay or buffer M for microscopy. Buffer B was the base buffer described in the main text (10 mM Tris pH 7.9, 100 mM NaCl, 50 mM KCl, 5 mM MgCl2, 0.1 mM EDTA) containing, in addition, 0.1 mg/mL BSA. For buffer M, the base buffer was supplemented with 5 mM DTT, 0.1 mg/mL dimethylated casein, 0.2% Tween-20, and 800,000-fold dilution of SYBR Gold. The reaction was started by the addition of 1 µL of the enzyme diluted in buffer B containing 0.5 mM DTT, and terminated by the addition of 2 µL of 5% SDS and 100 mM EDTA. Samples were mixed with 2 µL of gel loading buffer (50% glycerol, 0.9% SDS, 0.05% Bromophenol Blue), heated at 70°C for 2 min, subjected to electrophoresis in a 1% agarose gel in 90 mM Tris-borate pH 8.4 and 2 mM EDTA, and stained with 10,000-fold dilution of SYBR Gold. (A) Time courses of relaxation. (−)SC, negatively supercoiled plasmid; relaxed, relaxed circular plasmid. (B) Effects of topoisomerase-specific drugs. ICRF-193, a bis(2,6-didioxopiperazine) derivative, is a potent inhibitor of type II topoisomerase with 50% inhibition at ∼2 µM [16]. Etoposide inhibits religation of DNA, producing double-strand breaks irrespective of the presence of ATP [17]. Assays were made as in A for the reaction period of 30 min. Samples with etoposide were incubated, prior to electrophoresis, with 200 µg/mL proteinase K at 45°C for 30 min to digest topo IIα. Linear DNA marker was generated by digesting pBR322 by EcoRI. The yield of linearized DNA at 200 µM etoposide is below 10% at the enzyme/pBR322 molar ratio of five under similar conditions [17]. (TIF) [file pone.0034920.s001.tif]

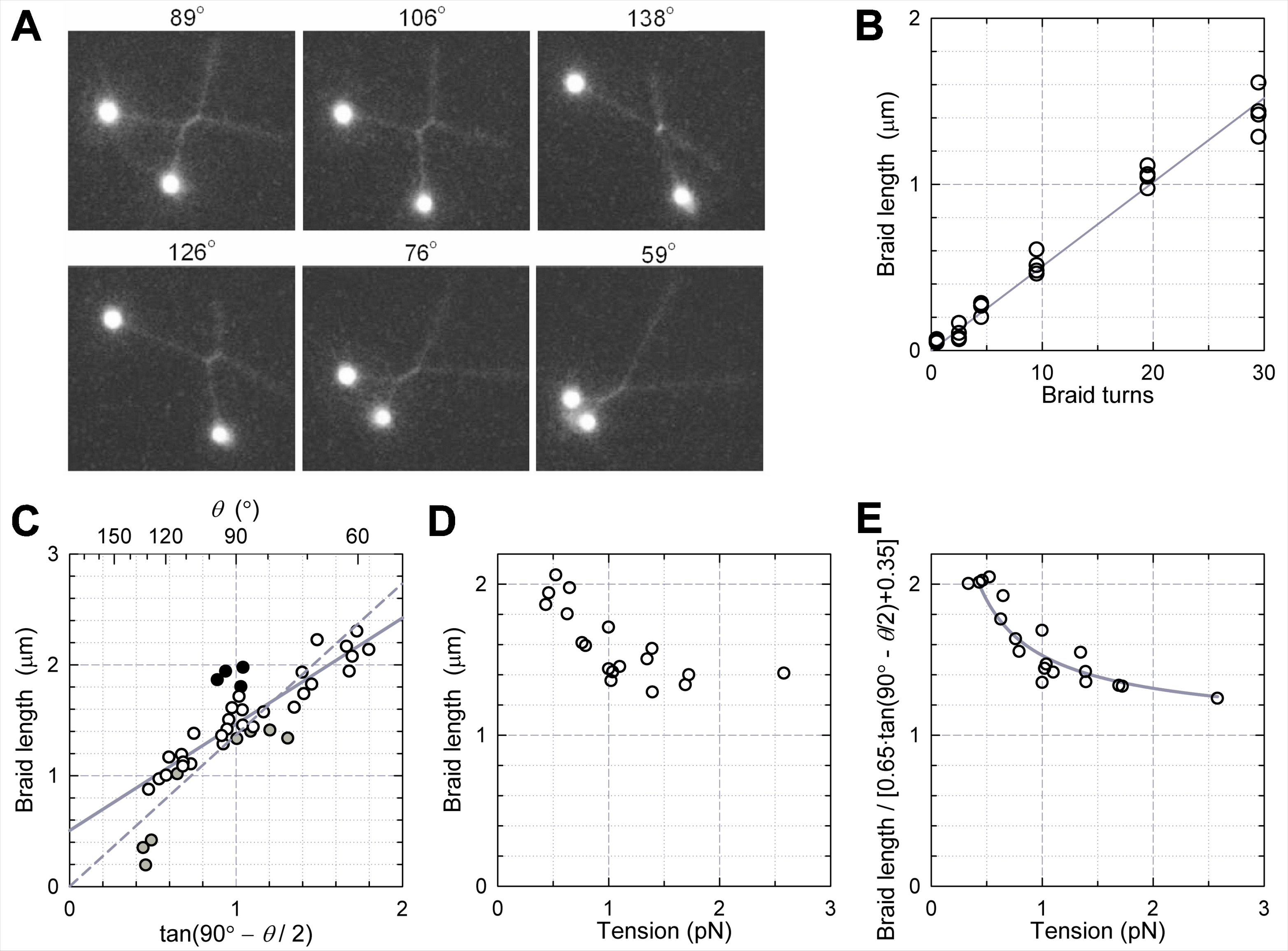

Supplement: Figure S3 — Determinants of braid length. Pairs of DNA were braided and the braid lengths estimated from fluorescence images as described in the main text, except that topoisomerase was not included in the medium. (A) Fluorescence images of a DNA braid of n = 30, showing dependence of the braid length on braid angles. (B) Proportionality between braid length and braid turns (n – 1/2) for θ around 90° (range 75°–95°). (C) Angle dependence of the braid length for n = 30. The lower horizontal axis is chosen to test the geometrical model in Figure S2. Open circles, tension between 0.7 and 1.4 pN; black circles, below 0.7 pN; gray circles, above 1.4 pN. Dashed line shows regression passing through the origin (geometrical model) for open circles, with l = 1.37tan(90° - θ/2) where l is the braid length in µm. A better fit is obtained if we allow the line to deviate from the origin (solid line), with l = 0.51+0.96tan(90° - θ/2). Deviation from the geometrical model (Figure S2) is expected for θ>90°, and the observed braid pitch of ∼50 nm (l/n), close to the persistence length of DNA, also suggests that bending the DNA may cost additional energy. (D) Tension dependence of the braid length (n = 30) for θ around 90° (range 79°–97°). (E) Braid lengths in D converted to those at θ = 90° by assuming the angle dependence shown in the solid line in C. Solid curve shows fit assuming that the normalized braid length depends on the tension F as F −3/4 [7]: l = 0.90+0.63F −3/4 where F is in pN. In the main text and in Figure S4 below, we estimate the braid turns n from the observed braid length l and tension F assuming this tension dependence and the solid line in C: l = [(n – 1/2)/(29.5·1.47)][0.51+0.96tan(90° - θ/2)][0.90+0.63F −3/4] or n = 1/2+72l/{[0.53+tan(90° - θ/2)](1.43+F −3/4)}. The root-mean-square deviation of the 58 measured braid lengths from this phenomenological equation (four data with tan(90° - θ/2)<0.5 excluded) is 0.12 µm, which is a measure of the reliability of the length esti [file pone.0034920.s003.tif]
