# Supplementary material for: Direct Observation of Strand Passage by DNA-Topoisomerase and Its Limited Processivity
Source: PLoS One. 2012 Apr 9;7(4):e34920. doi: 10.1371/journal.pone.0034920 (PMC3322154; doi:10.1371/journal.pone.0034920)
Supplement: Text S1 — DNA fluctuations in a braid. (DOC) [file pone.0034920.s006.doc]

**Text S1 DNA fluctuations in a braid.**

Here we present an order-of-magnitude estimation of the extent of DNA fluctuations in a braid. The treatment below is not rigorous nor precise, and numerical values should be regarded as such. The braids we consider are those in our experiments, where the four ends of a braid are pulled at nearly right angles under ~1 pN of tension.

First we consider longitudinal fluctuations of the middle of a 16-µm DNA held at its two ends. For DNA as a worm-like chain, the relation between its extension *L* and applied tension *F* can be approximated [23] by *FA*/*k*_B_*T* = (1/4)[(1 - *L*/*L*_0_)^-2^ - 1] + *L*/*L*_0_, where *L*_0_ is the contour length, *A* (~50 nm) the persistence length, and *k*_B_*T* (~4.1 pN⋅nm at room temperature) the thermal energy (*L*/*L*_0_ ~ 0.85 for *F* = 1 pN). When the two ends of a 16-µm DNA are fixed, its middle is constrained by two halves of the DNA each with a spring constant [24] *K*// = ∂*F*/∂*L*, which, for *L*_0_=8 µm and *F* = 1 pN in our experiment, is calculated as 1.5×10^-3^ pN/nm. The middle thus fluctuate, in the direction along the length of DNA, with an amplitude *Δz* characterized by <*Δz*^2^> = *k*_B_*T*/2*K*// ~ (37 nm)^2^.

In a DNA braid, the two DNA molecules are constrained laterally, but fluctuations of each DNA along the braid axis is not much restricted by the other DNA unless the tension is high enough to force the two DNAs physically touch with each other (*R* ~ 1 nm in Figure S2). Thus, the average amplitude of fluctuation of one DNA in a braid, along the braid length, will also be ~<*Δz*^2^>^1/2^ or ~37 nm. Fluctuations beyond this value are not rare: the probability of fluctuations beyond ±2<*Δz*^2^>^1/2^ ~ ±74 nm is ~5% and beyond ±3<*Δz*^2^>^1/2^ ~ ±110 nm is still as high as ~0.3%. The frequency of such fluctuations can very roughly be estimated as follows. The diffusion coefficient *D* of a 1-µm DNA segment is of the order of *k*_B_*T*/6*πηa* ~ 5×10^-13^ m^2^/s where *η* ~ 0.9×10^−3^ Ns/m^2^ is the viscosity of water and *a* = 0.5 μm (the dependence on the size, *a*, is modest and thus we take the length arbitrarily as 1 µm and adopt the simplest formula for *D*, for a sphere of radius *a*, which is an underestimate for the thin DNA segment). Free diffusion of the DNA segment over 74 nm thus takes (74 nm)2/2*D* ~ 0.005 s. When restricted by the rest of DNA as springs, diffusion over 74 nm = 2<*Δz*^2^>^1/2^ succeeds in ~5% of the cases, or once in ~0.1 s. Similarly, diffusion of the middle of DNA over 110 nm would take place once in ~4 s. Note that these frequency values are underestimated.

Thus, in the case of a simple DNA cross (a half-turn braid), topoisomerase does not have to bind precisely at the apex: even if it binds ±0.1 µm away, the binding site will fluctuate to the apex (or the other DNA will fluctuate to the binding site) more often than once a second. The effective target size for topo IIα binding is of the order of 0.2 µm in our experiments. In a long braid, a topo IIα molecule will continue to unbraid it until the topo IIα gets out of the braid zone by ~0.1 µm, as long as the topo IIα stays on the binding site on one DNA and remains active.

Next we consider lateral fluctuations of DNA. Suppose that a DNA of extension *L* is fixed at its two ends such that its tension is *F*. When its middle is displaced laterally by *Δx*, the two halves of DNA pull the middle back each with a force *F*·[*Δx*/(*L*/2)], or each with a spring constant [24] *K*_⊥_ = *F*/(*L*/2). Mean square displacement of the middle is given by <*Δx*^2^> = *k*_B_*T*/2*K*_⊥_. The pitch of our braid is ~50 nm (~1.5 µm / 30 turns; Figure S3), which is a measure of *L* above for lateral fluctuations. For *L* = 50 nm and *F* = 1 pN, <*Δx*^2^>^1/2^ ~ 7 nm. The two DNAs in our braid are separated laterally by an average distance of this magnitude (*R* in Figure S2). For lateral fluctuations of this size, DNA may be considered to consists of segments of length *A* (persistence length) that move relatively independently, with a diffusion coefficient an order of magnitude larger than the above *D* of 5×10^-13^ m^2^/s. Lateral encounters of two DNAs in a braid should thus be frequent, well above a thousand times a second (depending on how an encounter is defined).
